# Aggressive NK-Cell Leukemia

**DOI:** 10.3389/fped.2018.00292

**Published:** 2018-10-10

**Authors:** Fumihiro Ishida

**Affiliations:** Department of Biomedical Laboratory Sciences, Shinshu University School of Medicine, Matsumoto, Japan

**Keywords:** NK cell, L-asparaginase, JAK/STAT, allogeneic hematopoietic cell transplantation, Epstein-Barr virus, LGL

## Abstract

Aggressive NK cell leukemia (ANKL) is a rare malignant lymphoproliferative disorder of mature NK cells closely associated with Epstein-Barr virus (EBV) and more common in East Asia than in other areas. Significant variations exist in the morphology of ANKL tumor cells, from typical large granular lymphocyte morphology to highly atypical features with basophilic cytoplasm containing azurophilc granules. The main involved sites are hepatosplenic lesions, bone marrow and peripheral blood, and nasal or skin lesions are infrequent. A fever and liver dysfunction with an often rapidly progressive course are the main clinical symptoms, including hemophagocytic syndrome and disseminated intravascular coagulation. Although the outcome had been dismal for decades, with a median survival of less than three months, the introduction of combined chemotherapy including L-asparaginase and allogeneic hematopoietic cell transplantation has helped achieve a complete response and potential cure for some patients. With the advent of next-generation sequencing technologies, molecular alterations of ANKL have been elucidated, and dysfunctions in several signaling pathways, including the JAK/STAT pathway, have been identified. Novel target approaches to managing these abnormalities might help improve the prognosis of patients with ANKL.

## Introduction

Aggressive NK cell leukemia (ANKL) is a rare malignant lymphoproliferative disease of mature NK cell type (1–3). ANKL is prevalent among Eastern Asian populations compared with Western countries and develops mainly in the relatively young (4–6). ANKL is closely associated with Epstein-Barr virus (EBV) (7, 8), with only 10% of ANKL cases negative for EBV (9). With the advent of next-generation sequencing technologies, the molecular basis of ANKL has been considerably elucidated. However, the prognosis of ANKL is still quite poor, with a median survival duration shorter than one year (6, 10). I will discuss several molecular and clinical issues associated with ANKL in this review.

## Terminology

ANKL has been categorized as a distinct entity since the third WHO classification and has been also classified as a large granular lymphocyte (LGL) leukemia based on its morphological features (3, 11). ANKL is further mentioned as a leukemic type of mature NK cell lymphoproliferative disorder, another type of which was designated chronic lymphoproliferative disorders of NK cell (CLPD-NK) in WHO 2017 classification, provisionally. ANKL is also discussed in relation to NK cell neoplasms, especially extranodal NK/T cell lymphoma, nasal type (ENKL), based on its immunophenotype.

It is thus appropriate that ANKL be classified as an EBV-associated mature malignant NK cell neoplasm and discussed in special relation to ENKL and/or EBV-positive T cell and NK-cell lymphoproliferative disease of childhood, rather than indolent LGL leukemia, such as T cell LGL leukemia or CLPD-NK, based on the current molecular and clinical recognition of ANKL.

## Epidemiology

**Figure 1:**
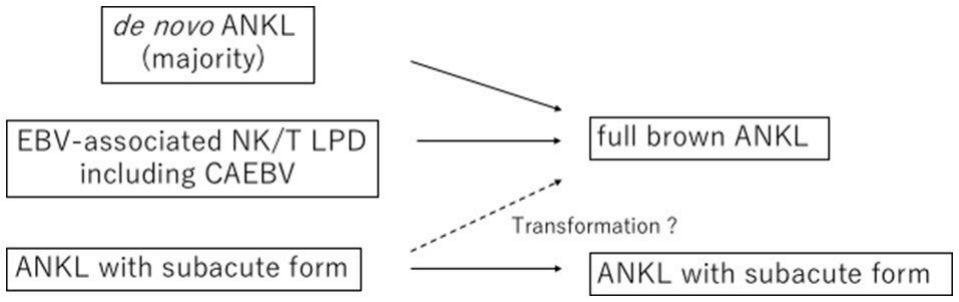
Development of aggressive NK-cell leukemia (ANKL). Most of aggressive NK-cell leukemia (ANKL) develops in *de novo* form and in some younger patients, ANKL evolves from EB virus-associated NK/T cell lymphoproliferative disorders including chronic active EB virus infection (CAEBV) disease. Subacute form of ANKL has been also reported, although it is uncertain whether subacute ANKL transforms into full brown ANKL.

ANKL develops mainly in patients between 20 and 50 years of age but has also been reported in teenagers and patients in their 70s (5, 6, 12). Transformation into ANKL from EBV-associated NK/T lymphoproliferative disorders, such as chronic active EBV infection, is particularly prone to occur in patients of younger age (13, 14) (Figure 1). Therefore, ANKL should be recognized as a malignancy among adolescent and young adult (AYA) populations. Approximately 400 cases have been reported, with an increasing number of patients being reported from Caucasian populations and Latin American areas (4–6, 12, 15–20). Whether or not clinical characteristics differ geographically or with ethnic backgrounds is unclear at present.

## Molecular pathogenesis

Genetic alterations of ANKL have been largely unclear, in contrast to the wealth of information available for ENKL, a closely related disease of ANKL. With target sequencing of a limited number of ANKL patients, including EBV-negative cases, mutations in *STAT3* or *STAT5B*, molecules of the JAK/STAT signaling system, have been identified, but not in *JAK3*, the molecule in which recurrent mutations were first identified in ENKL (18, 21, 22).

Two groups recently performed a comprehensive genetic analysis of ANKL with next-generation sequencing technology (Supplementary Table). Dufva et al. analyzed 14 patients of ANKL with whole-exome sequencing (23). The median number of non-synonymous somatic mutations was 105. Frequent genetic mutations were recognized in the signal transduction system, including the JAK/STAT and RAS-MAPK systems. In 21% of cases, *STAT3* was mutated. In approximately half of the cases, mutations in epigenetic regulatory molecules and or histone modification molecules were also detected, including four cases with *DDX3X*, an RNA helicase. Huang et al. analyzed 8 patients with ANKL by whole-genome sequencing and 29 patients by target sequencing (24). The mean number of non-synonymous mutations was 40. In 48% of cases, mutations in molecules of the JAK-STAT system were detected almost mutually exclusively, and *STAT3* mutations were the most frequent at 17%. In epigenetic modification-related genes, *TET2* (28%), *CREBBP* (21%) and *MLL2* (21%) mutations were found. *TP53* mutations were also recognized in 34% of cases. In contrast, *DDX3X* and *BOCR* were less frequently mutated. Because of the high frequency of mutations in the JAK-STAT system, the authors examined the levels of inflammatory cytokines and found that the plasma IL-10 levels were significantly elevated in ANKL patients and that activation of the JAK/STAT system in ANKL led to an increased expression of MYC, implying the importance of IL-10-STAT3-MYC transcription regulation in ANKL.

Certain chemokine receptors expressed on ANKL cells, especially CXCR1 and CCR5, might be associated with organ damage, including liver damage, in ANKL along with corresponding chemokines (25). Indeed, the serum levels of IL-8, MIP-1α, and MIP1β were significantly elevated in ANKL (26). Interferon γ is also an important regulator in ANKL (27, 28).

## Is ANKL the same as advanced-stage ENKL?

There has been debate regarding the relationship between ANKL and advanced-stage ENKL—namely, whether or not ANKL is a leukemic form of ENKL—since several common features exist between these two diseases, such as their cellular phenotypes and close association with EBV. Furthermore, almost the same therapeutic strategies are currently applied for these diseases.

### Genetic abnormalities

An earlier study reported on the genetic differences between ANKL and ENKL. An array comparative genomic hybridization analysis showed that gain of 1q23.1-q23.2 and 1q31.3-q44 and loss of 7p15.1-q22.3 and 17p13.1 were more frequently recognized in ANKL than in ENKL (29). Furthermore, the loss of 6q16.1-q27, which was reported as a common region in NK-cell malignancies in a previous study (30), was frequently recognized in ENKL but less so in ANKL (29). On next-generation sequencing, ANKL showed a different mutation signature from ENKL, with fewer TP53 mutations and more mutations in RAS-MAPK signaling pathway genes, although several common mutations with ENKL were identified, and the frequencies of *TP53* mutations differed (23).

### Biological aspects

An immunophenotypic analysis showed that the CD16 expression on tumor cells was positive in 75% of ANKL cases, but only in 22% of ENKL cases (31). Commonly involved sites were the nose and skin in ENKL and the liver, spleen, peripheral blood and bone marrow in ANKL (31), in which the disease definitions including lesions of these two diseases might reflect.

Granulysin, a cytotoxic molecule in the cytotoxic granules of NK cells, can be detected in the sera. The granulysin levels were significantly elevated in ANKL compared to stage IV ENKL and normal subjects (32).

## EBV-negative ANKL

ENKL is essentially positive for the EBV genome in all cases. In contrast, a certain proportion of ANKL patients (~10%) are negative for EBV. Initial reports have suggested a better prognosis of EBV-negative ANKL than EBV-positive ANKL (9), but findings are not consistent (6, 18). No morphologic or immunophenotypic features reliably discriminate between EBV-positive and EBV-negative ANKL (9, 18).

## The diagnosis of ANKL

The diagnosis of ANKL should be made based on three factors: the cellular characteristics, involved sites, and clinical features (5, 6). The tumor cells morphologically resemble large granular lymphocytes and are sometimes pleomorphic and large and immunophenotypically CD2+ surface CD3-, CD3epsilon+, CD16+, and CD56+, with a lack of myeloid and B-cell markers. The T-cell receptor genes are in a germline configuration, and the EBV genome is usually positive. The main involved sites are the bone marrow, peripheral blood, liver, and spleen. The clinical features consist of a fever, hemophagocytosis, liver dysfunction, disseminated intravascular coagulation and a progressive course of weeks or sometimes months. In some patients, the diagnosis of ANKL has been delayed and challenging because of the presence of non-specific clinical symptoms and fewer tumor cells in the peripheral blood and/or bone marrow on presentation. Appropriate diagnostic methods for ANKL must be established.

## Do some ANKL patients show a different clinical time frame from others?

Some patients with ANKL are known to present with indolent characteristics and later develop aggressive disease or have a slowly progressive course (33). Tang et al. defined the subacute form of ANKL as ≥90 days of infectious mononucleosis-like symptoms, and 16% of their patients corresponded to this form (17). They compared the gene mutation profiles between the subacute forms and the other patients and found that the patients with the subacute form possessed fewer TP53 mutations and had a better prognosis than those with the typical form, which further implies the heterogeneity of ANKL.

## Chemotherapy for ANKL

The optimum chemotherapy regimen as an initial treatment for ANKL has not been established. There have been no prospective clinical trials conducted solely for ANKL. With anthracycline-containing chemotherapy, some patients have achieved a complete response (5). *In vitro*, ANKL cell lines and tumor cells from these patients were shown to be sensitive to L-asparaginase, leading to apoptosis (34). Furthermore, an improved outcome for ANKL with L-asparaginase-containing chemotherapy has been shown (6). Subsequently, the significant efficacy of L-asparaginase against ANKL has been recognized (10, 21). Treatments with L-asparaginase have contributed to an improved survival. Various L-asparaginase-containing regimens, such as SMILE (dexamethasone, methotrexate, ifosfamide, etoposide, and L-asparaginase), AspaMetDex (L-asparaginase, methotrexate, and dexamethasone) or VIDL (etoposide, ifosfamide, dexamethasone, and L-asparaginase) in addition to L-asparaginase monotherapy have been utilized, although no study has compared these regimens for ANKL (10, 35, 36). The formulation of L-asparaginase—as native *E. coli* asparaginase, pegylated-asparaginase or *Erwinase* asparaginase—has also varied. A report implied the effectiveness of gemcitabine, cisplatin, and dexamethasone (GDP) in selected patients (37). A complete response, including negativity for EBV DNA in the blood after treatments, was associated with a better outcome, including the overall survival (10). However, even patients who achieved a CR after chemotherapy including L-asparaginase rarely survived for more than one year without further treatments, especially allogeneic hematopoietic cell transplantation (HCT) (6, 10, 38).

Several prognostic factors for ANKL, such as the patient age, serum lactose dehydrogenase levels and serum total bilirubin level (17), have been proposed, but none have been validated.

## Role of hematopoietic cell transplantation for ANKL

Although patients have responded to chemotherapy including L-asparaginase, almost all patients eventually died of their disease. Therefore, in order to improve the outcome, hematopoietic cell transplantation has been applied in select patients with ANKL (35, 39, 40). Among the eight patients with a non-CR condition before HCT who received allogeneic HCT, four reached CR, and two of them survived for several years (6). Subsequent studies have also shown the significant efficacy of allogeneic HCT for ANKL (10, 17). A total of 21 ANKL patients registered between 2000 and 2014 in the International Bone Marrow Transplantation Registry (IBMTR) database underwent allogeneic HCT, with most receiving L-asparaginase-containing chemotherapy before proceeding to HCT (38). Patients with a CR prior to HCT showed a significantly better survival after two years than those without a CR (38 vs. 0%). However, 76% of all patients died in the long run, mostly due to ANKL.

Allogeneic HCT for ANKL might help extend the survival, including achieving a cure, in some patients, but the success is limited. Myeloablative conditioning regimens were used in most cases, and the use of non-myeloablative regimens has been increasing. The ideal donor source has not been defined, but performing HCT as early as possible has been suggested to lead to a better outcome in some studies (6, 38), which might be worth considering. The role of autologous HCT in ANKL is uncertain. Given the retrospective nature of studies on HCT for ANKL, selection bias might also affect these results.

## Developments of new therapies for ANKL

### Immune checkpoint inhibitors

Immune checkpoint inhibitors, which have been widely applied in various malignancies and shown to be highly efficacious, were also demonstrated to be effective in patients with ENKL (41, 42). These patients were resistant to multiple chemotherapies, including L-asparaginase, and had very limited therapeutic options with a short life expectancy. Among the 10 patients with refractory or relapsed ENKL who were resistant to L-asparaginase-containing therapy, a 50–100% response to pembrolizumab or nivolumab was observed, including a CR. No unexpected adverse effects were recognized. While these were retrospective and small-sized studies, it would be of interest to examine the efficacy of immune checkpoint inhibitors in ANKL, a disease very closely related to ENKL.

### Other candidate agents

Several novel agents have shown significantly efficacy against ANKL cell lines *in vitro*. Decitabine, a hypomethylating agent, and vorinostat, an HDAC inhibitor, showed significant suppressive effects on select cell lines (43). A panel of drug sensitivity and resistance testing showed the JAK inhibitor ruxolitinib and the BCL2 inhibitor navitoclax to be highly effective against malignant NK cell lines. In addition, synergic relationships were observed between ruxolitinib and the BCL2 inhibitor venotoclax or the aurora kinase inhibitor alisertib, which further supports the notion that JAK-STAT alteration is a potential therapeutic approach to ANKL (23). Most of these drugs have already been approved for the treatment of other hematological malignancies, and their efficacy against ANKL must be proven in future studies before they can be applied in clinical practice.

## Conclusions

Significant progress has been made in understanding the molecular pathogenesis and clinical characteristics of ANKL; however, the outcomes of ANKL patients remain poor. Novel approaches, including targeted therapy, such as that for JAK-STAT signaling systems, and immune therapy, such as immune checkpoint inhibitors, HCT and cellular immune therapy with CAR-T cells, or combinations of these approaches may help improve the prognosis of this devastating disease.

## Author contributions

The author confirms being the sole contributor of this work and has approved it for publication.

### Conflict of interest statement

The author declares that the research was conducted in the absence of any commercial or financial relationships that could be construed as a potential conflict of interest.
